# Supplementary material for: Design, development, and evaluation of gene therapeutics specific to KSHV-associated diseases
Source: Mol Ther Oncol. 2025 Sep 5;33(4):201050. doi: 10.1016/j.omton.2025.201050 (PMC12481920; doi:10.1016/j.omton.2025.201050)
Supplement: Document S1. Figures S1–S4 and Table S1 [file mmc1.pdf]

OMTON, Volume *33*

Supplemental information

Design, development, and evaluation of gene therapeutics specific to KSHV-associated diseases

Tomoki Inagaki, Jonna Magdallene Espera, Kang-Hsin Wang, Somayeh Komaki, Sonali Nair, Ryan R. Davis, Ashish Kumar, Ken-ichi Nakajima, and Yoshihiro Izumiya

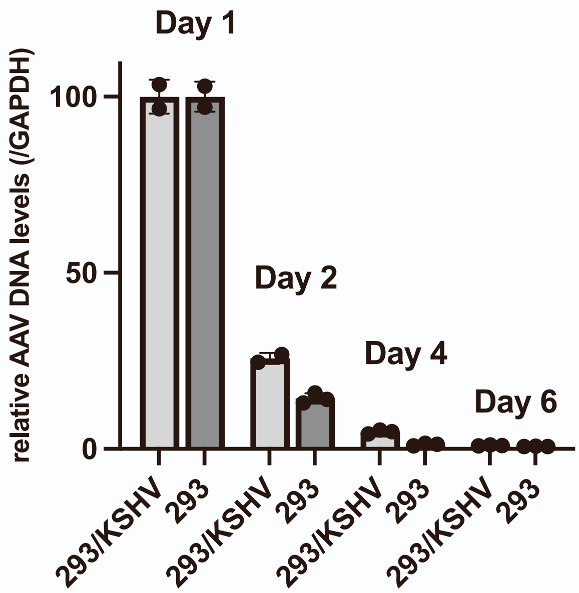

Figure S1. Relative AAV DNA copy number in KSHV infected or non-infected 293T cells for 6 days. AAV DNA copies were measured by qPCR and compared between the 293/KSHV and the parental 293 cells. Twenty-four hours after AAV-mCardinal transduction in 293/KSHV cells was designated as 1. GAPDH coding sequence was used for internal control. Data was analyzed using a two-sided unpaired Student's t-test and shown as mean ± SD.

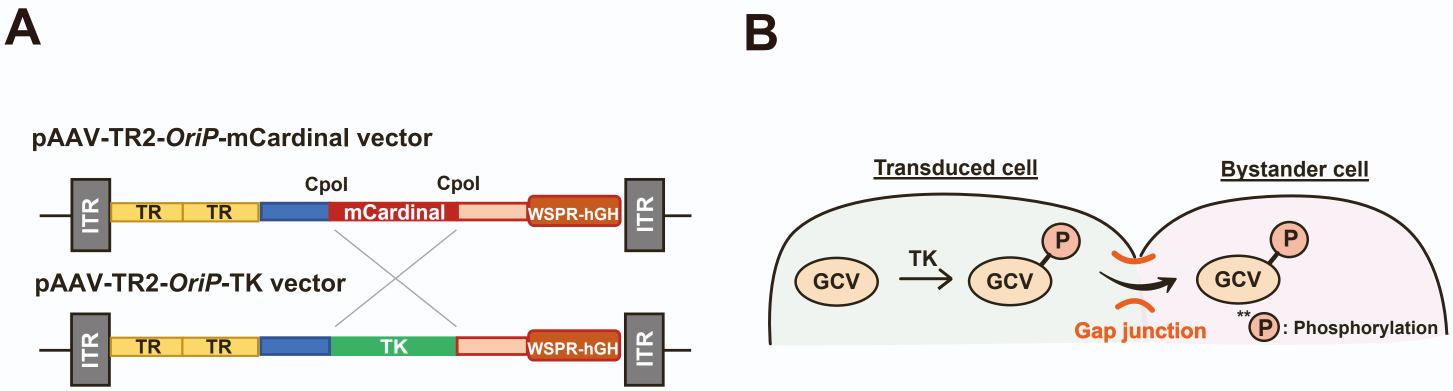

Figure S2. Construction of pAAV-TR2-*OriP*-TK vector. A. Schematic diagram of pAAV-TR2-*OriP*-TK vector. mCardinal sequence encoding in the pAAV-TR2-*OriP*-mCardinal vector was replaced with TK sequence. TR: terminal repeat, TK: thymidine kinase, ITR: inverted terminal repeat. **B. Schematic diagram of TK/GCV system.** TK phosphorylates the prodrug ganciclovir (GCV) into a toxic nucleotide analog, leading to selective cell death in TK-expressing cells. The phosphorylated GCV can also diffuse into neighboring bystander cells by gap junction, inducing cytotoxic effects even in non-TK-expressing cells

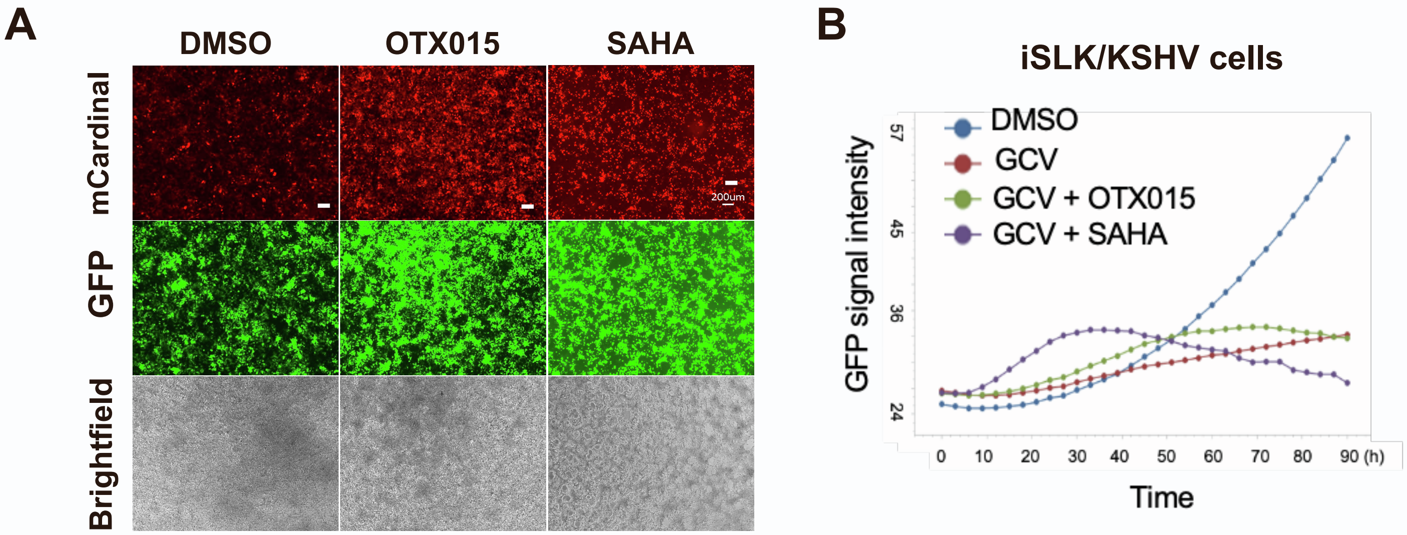

Figure S3. SAHA and OTX015 stimulate transcription from the TR2-*OriP* vector. a. Fluorescent and bright field cell images. KSHV-infected 293 cells were seeded in 12 well plates and transduced with AAV8-TR2-*OriP*-mCardinal. Two days after AAV8-TR2-*OriP*-mCardinal infection, cells were treated with mock (DMSO), OTX015 (200 nM), or SAHA (1 μM). Images were taken four days after the AAV infection. Scales: 200 μm**. b. KSHV-infected 293 cell growth.** KSHV-infected 293 cells were seeded in 6 well plates, and GCV (10 μg/ml) with or without OTX015 (200 nM) or SAHA (1 μM) were added to cells two days after AAV8-TR2-*OriP*-TK infection. Cell growth (upper) and GFP signal intensity (lower) were continuously monitored by Incucyte for 90 hours.

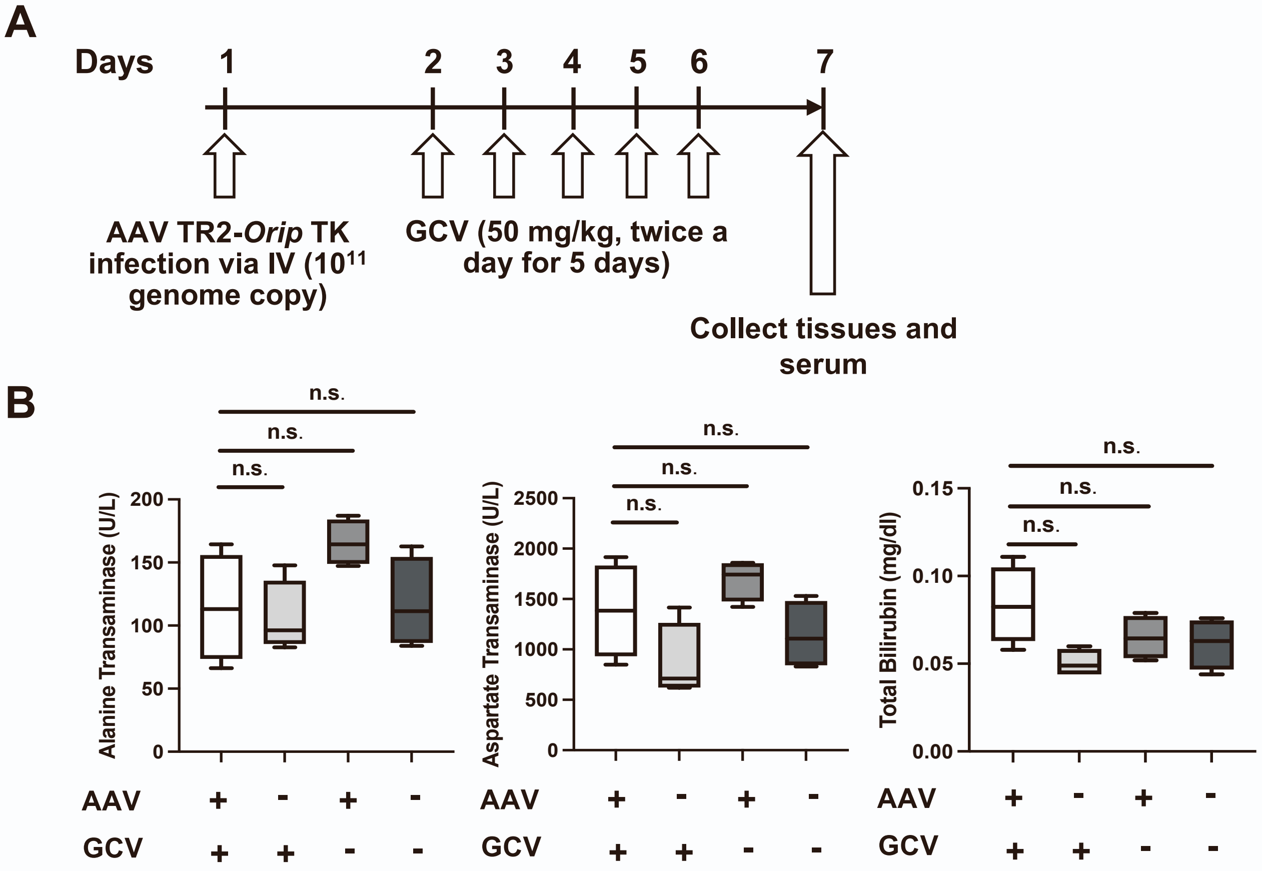

Figure S4. A. Schematic diagram of toxicity studies. B. AAV-8 TR2-*OriP*-TK showed no liver cell cytotoxicity. The levels of liver enzymes (U/L) and total bilirubin (mg/dl) after systemic AAV-infection with GCV. Serum samples were collected 24 hours after last GCV administration.

Table S1

Primers name	Direction	Sequence 5'-> 3'
PAN-RNA	forward	5'- GTTTCGGTTCTGTGTTTGTCTG
	reverse	5'-CACAACGCACCAATAAGATACAC
K8.1	forward	5'-AAAGCGTCCAGGCCACCACAGA
	reverse	5'-GGCAGAAAATGGCACACGGTTA
ORF6	forward	5'-CTGCCATAGGAGGGATGTTTG
	reverse	5'-CCATGAGCATTGCTCTGGCT
CD34	forward	5'-CCTCAGTGTCTACTGCTGGTCT
	reverse	5'-GGAATAGCTCTGGTGGCTTGCA
Prox-1	forward	5'-CTGAAGACCTACTTCTCCGACG
	reverse	5'-GATGGCTTGACGTGCGTACTTC
Flt-4	forward	5'-TGCGAATACCTGTCCTACGATGC
	reverse	5'-CTTGTGGATGCCGAAAGCGGAG
LYVE-1	forward	5'-GCCGACAGTTTGCAGCCTATTG
	reverse	5'-CCGAGTAGGTACTGTCACTGAC
Oct3/4	forward	5'-ATTCAGCCAAACGACCATC
	reverse	5'-GGAAAGGGACCGAGGAGTA
Nanog	forward	5'-CAGCCCTGATTCTTCCACCAGTCCC
	reverse	5'-TGGAAGGTTCCCAGTCGGGTTCACC
Sox2	forward	5'-CAGCGCATGGACAGTTAC
	reverse	5'-GGAGTGGGAGGAAGAGGT
18S rRNA	forward	5'-TTCGAACGTCTGCCCTATCAA
	reverse	5'-ATGGTAGGCACGGCGACTA
GAPDH	forward	5'-TCGCTCTCTGCTCCTCCTGTTC
	reverse	5'-CGCCCAATACGACCAAATCC
Ori-RNA promoter SpeI	forward	AAAACTAGTGCATCGCAGCCCCTATTCCAGTAGGTATAC
Ori-RNA promoter KpnI-BamHI	reverse	AAAGGTACCGGATCCTTATCGATTTTACCACATT
AAV cloning sites S	forward	CGGAATTCGCCCTTAAGCTAGCTTCTAGACGGTACCAGGATCCAATCAACCTCTGGAT
AAV cloning sites AS	reverse	ATCCAGAGGTTGATTGGATCCTGGTACCGTCTAGAAGCTAGCTTAAGGGCGAATTCCG

Codon optimized fragment		Sequence
TKSR39		TCCGGTACTGTTGGTAAAGCCGGTCCGACCATGGCATCTTACCCTTGCCATCAGCACGCGTCAGCGTTCGACCAAGCTGCTAGGAGCCGCGGACACAACAACAGAAGAACGGCATTGCGGCCGAGACGGCAGCAAAAGGCAACTGAGGTCCGCTTGGAACAAAAAATGCCCACGCTGCTTCGAGTCTATATTGACGGTCCTCATGGAATGGGCAAGACTACTACGACTCAGCTGTTGGTCGCACTTGGCTCAAGAGATGATATAGTGTATGTGCCCGAGCCAATGACTTACTGGCGCGTTCTTGGCGCTAGTGAGACTATCGCCAATATTTACACGACACAACACAGACTGGACCAGGGAGAGATTTCTGCGGGAGATGCAGCGGTAGTGATGACCTCTGCGCAGATTACAATGGGAATGCCGTACGCCGTTACCGACGCGGTTTTGGCACCTCACATAGGAGGAGAGGCAGGGTCTTCCCATGCCCCTCCTCCCGCCCTCACAATTTTTCTTGACCGCCATCCAATAGCTTTTATGCTGTGCTACCCCGCTGCGAGGTACCTGATGGGCAGTATGACTCCCCAAGCAGTGCTTGCATTCGTAGCTCTGATACCACCTACACTGCCGGGGACGAATATCGTCCTGGGGGCGCTTCCAGAAGATAGGCATATTGATAGATTGGCAAAACGCCAACGCCCCGGAGAGCGCCTTGACTTGGCTATGCTCGCTGCGATCAGACGCGTGTACGGCTTGCTCGCCAATACTGTCAGGTATCTCCAAGGTGGTGGCAGCTGGAGGGAAGATTGGGGACAATTGTCCGGCGCGGCCGTGCCCCCTCAAGGAGCTGAACCGCAGTCCAACGCCGGGCCGAGGCCCCATATAGGGGACACACTCTTTACTCTGTTTAGAGCACCTGAGCTGCTGGCGCCTAACGGAGATCTTTACAACGTGTTCGCGTGGGCGTTGGATGTGCTTGCAAAAAGGCTCCGACCGATGCACGTTTTCATTCTGGATTATGACCAGTCCCCTGCTGGGTGCCGCGACGCACTCCTTCAACTCACATCAGGGATGGTGCAAACTCACGTAACTACCCCAGGGAGCATACCTACTATCTGCGATCTTGCTCGGACATTTGCACGAGAGATGGGGGAGGCCAATTAACGGTCCGAATCAACCTCTGGATTACAAA

List of Movies for iSLK cells growth. Images were taken every 3 hours continuously by Incucyte for four days.

Video S1. Cells were treated with mock,
Video S2. GCV (5 μM),
Video S3. GCV and OTX015 (200 nM)
Video S4. GCV and SAHA (1 μm)
